# Supporting Women’s Leadership Development in Global Health through Virtual Events and Near-Peer Networking

**DOI:** 10.5334/aogh.3397

**Published:** 2022-01-07

**Authors:** Anna Kalbarczyk, Meagan Harrison, Eumihn Chung, Nancy Glass, Becky Genberg, Michele R. Decker, Yukari C. Manabe

**Affiliations:** 1 Johns Hopkins Center for Global Health, Baltimore, USA; 2 Johns Hopkins Bloomberg School of Public Health, Department of International Health, Baltimore, USA; 3 Johns Hopkins School of Nursing, Baltimore, USA; 4 Johns Hopkins Bloomberg School of Public Health, Department of Epidemiology, Baltimore, USA; 5 Johns Hopkins Bloomberg School of Public Health, Department of Population, Family, and Reproductive Health, Baltimore, USA; 6 Johns Hopkins School of Medicine, Baltimore, USA

## Abstract

Women leaders have gained increasing attention during the COVID-19 pandemic for their positive influence on health and unique abilities to manage a global crisis, but women continue to face significant barriers to reaching and maintaining leadership positions. We developed a virtual leadership program to promote the experiences of women leaders in global health in different disciplines and career paths to develop concrete recommendations for young women and their institutions. This program includes a speaker series, interactive working groups, and a near-peer networking platform. In 2020, five global leaders spoke to over 1,300 unique attendees representing 44 countries and shared their leadership experiences and key lessons learned. Leaders urged young women to take advantage of unexpected opportunities rather than follow discrete plans; build bridges with each other to foster diversity and inclusion; find their passions; and bolster ‘essential skills’ (i.e., communication and self-awareness). A brief online survey was circulated after each event. Seventy-nine percent of respondents (n = 158) agreed or strongly agreed that they have a greater understanding of solutions to combat challenges that women face in global health leadership and 54% (n = 107) of respondents reported that the event strengthened their network. The virtual approaches employed by this program in combination with the pandemic lockdown likely provided a unique opportunity to recruit high-level speakers and focus financial resources on communication and outreach. This type of programming can support a diverse cadre of women leaders including those with intersecting identities that are often marginalized or historically invisible in leadership ranks.

## Introduction

Women leaders have gained increasing attention during the COVID-19 pandemic for their positive influence on health and unique abilities to manage a global crisis. In May, 2020 the New York Times published an article, ‘Why are Women-Led Nations Doing Better with Covid-19?’ highlighting lessons we can learn from women’s leadership styles such as inclusion of diverse perspectives when making complex decisions [1]. In 2019, McKinsey published a report highlighting that women, more frequently than men, exhibit leadership traits highly applicable to future global challenges such as inspiration and participative decision making [2]. As with their male counterparts, women’s leadership cannot be distilled down to a singular strategy or style. Moreover, the leadership traits for which they may be best recognized, e.g., inspiration and participatory decision-making, likely reflect adaptive strategies born of necessity as women successfully navigate organizational and social cultures with embedded gender hierarchies that historically marginalize women’s leadership and relative power in decision-making, social, economic and political.

Women continue to face significant barriers to reaching and maintaining leadership positions [3]. It has been widely cited that women comprise as much as 75% of the health workforce in many countries but are underrepresented in leadership positions [4]. Yet, when given the opportunity (as we have seen during the COVID-19 pandemic), women leaders can yield remarkable impacts. Studies in India and Afghanistan show that districts with women-elected officials have documented better health and education outcomes [5]. A recent study assessed the impact of leaders on environmental performance in the financial industry (banks, specifically) and found that gender diversity in leadership is an important driver of environmental sustainability [6]. Findings from a meta-analysis of 87 independent samples taken from over 20 countries, including low-and middle-income countries (LMICs), show that female board representation increases a firm’s engagement in corporate social responsibility and leads to more favorable social reputations [7]. The value of women’s leadership globally cannot be overstated.

There are many leadership theories, models, and frameworks that have been applied specifically to women’s leadership recognizing that women tend to exhibit different leadership traits than men [8]. In 2011, Ely et al. argued that, like the styles and traits that women exhibit in leadership, development activities are also unique and efforts to develop women leaders must be tailored to them [9]. For example, mentorship is a key component of many leadership development programs but historically women were paired with male mentors (since men hold most leadership positions). Networks are also vital instruments in leader development but not all networks are created and used for the same purposes. Specifically, men’s professional networks tend to be used for practical reasons related to job and career while women’s networks tend to be leveraged for relationship building and support [10]. The role model effect, the actual visibility of role models in the work environment, may be vital to produce empowerment effects for future generations of women leaders. Beyond being inspirational, women role models show other women how to be and act in challenging work situations. Specific to leadership, women actively mimic their role model’s powerful nonverbal behaviors (e.g., body postures), which can lead to confidence when taking leadership actions that then leads to enhanced performance in their own work environment [11]. We developed a virtual event series, informed by the role model effect, to promote the experiences of women leaders in global health in different disciplines and career paths and develop concrete recommendations for young women and their institutions to improve approaches and opportunities to leadership. Our approach sought to tailor common leadership development strategies for young women by creating spaces that could be used for reflection, relationship development, and career advancement.

This paper describes the development of this series, its evolution during the COVID-19 pandemic, an emergent near-peer network, and key recommendations for institutions and individuals to support women leaders in global health moving forward. The secondary analysis of programmatic data was approved by the Johns Hopkins Bloomberg School of Public Health Institutional Review Board.

## A virtual series on women’s leadership

In January 2020, the Johns Hopkins University (JHU) Provost’s Office launched a PhD Professional Development Innovation Initiative, which seeks to ensure that JHU doctoral students, while immersed in their training, have exposure to and the opportunity to explore a wide range of career options [12]. Through this initiative, JHU faculty were invited to submit proposals to develop events that would facilitate this outcome.

Our approach was designed to expose doctoral students at JHU and among other partner institutions to global leaders in non-academic careers, with a lens that centered on their varied approaches to leadership. We conceptualized the events using a collaborative process that purposefully incorporated the voices of women in different disciplines within global health (i.e., medicine, nursing, epidemiology, etc.) and at different stages of their careers.

The initial proposal was developed prior to the COVID-19 pandemic and included a series of 6 in-person keynote speakers who would provide a 30-minute talk followed by a moderated question and answer session and subsequent working group event. During the working group, participants were asked to consider the lessons shared by the speaker, reflect on their own experiences, and develop recommendations and solutions for individuals, institutions, and academic programs. This would create a solutions-oriented environment and allow participants additional time to get to know each other and grow their network. The keynote would be live streamed online to allow for greater participation from non-JHU students and students on other campuses.

As with most programming in 2020, the pandemic—within the context of a global anti-racism movement led by Black Lives Matter (BLM)—pushed us to not only rethink our design and revise our delivery but recognize that academic institutions, international development, and nonprofit sectors have a significant history of racist and gendered power dynamics. Importantly, women of color working within international organizations, including those with women leadership, began to share their stories of the racist systems and structures that limited their opportunities for career and professional advancement [13].

While all activities were transferred online, key components remained the same: we recruited racial and ethnically diverse women leaders with diverse cultural backgrounds and career paths to serve as speakers and actively recruited JHU and partner institution students to join the events. We maintained the working group component though altered it to be conducted as a stand-alone event, following the leadership talks. More details about these activities can be found on the Initiative’s website [14]. What is clear is that we as an organization and as individuals have work to do.

## Lessons from our Leaders

From January–December 2020 we hosted 5 virtual seminars with prominent women leaders in global health including Dr. Leslie Mancuso, CEO of Jhpiego; Dr. Madeleine Albright, Former US Secretary of State; Dr. Agnes Binagwaho, Former Minister of Health Rwanda; Dr. Sheila Davis, CEO of Partners in Health; and Dr. Lola Adeyemi, CEO and Founder of Mentoring Her. Each speaker was asked to discuss their leadership journey and provide lessons learned for the audience. Recordings of each talk are available freely online [15]. Common themes emerged across the five talks:

### Taking advantage of unexpected opportunities

Each speaker described the importance of assessing and seizing opportunities throughout their careers and encouraged attendees to do the same. Dr. Davis called this the Spider-Man theory of change leadership and said, ‘follow your passion, not your path.’ She further described how well-planned paths might prevent young professionals from growing in unexpected ways. Dr. Albright echoed this sentiment, noting that ‘accidental good things’ have expanded her horizons and made her more valuable throughout her career which in turn opened additional professional leadership opportunities.

Dr. Mancuso further highlighted the importance of life-long learning, particularly in diverse professional areas. She described spending additional time to acquire the skills she needed as she grew into leadership roles; for example, time with financial teams to learn the complexities of financial management and reporting, a key skill needed in her CEO role. Dr. Davis noted that when she was interviewed for her current leadership position, she was honest about which skills she did and did not possess; this provided opportunities for learning and networking with those who could best support her.

As a social entrepreneur, Dr. Lola Adeyemi told participants not to let perfection get in the way of progress. That is, once you have an idea, go for it; but she also recognized the impact of burnout and needing to take a step back. She poignantly said, ‘setbacks should be opportunities for comebacks,’ encouraging participants to take breaks and focus on self-care when needed.

### Building bridges, not dams

The notion of supportive people and supportive environments carried throughout the series. Dr. Binagwaho focused on a collective spirit of women’s leadership, working with both men and women to ensure that women are equally represented in the field. She noted that having more women in global health leadership will change the situation of women globally. Dr. Mancuso similarly reflected that we can only move countries forward if women are part of the solutions and their development. Dr. Binagwaho also emphasized the role of intersectionality in this solution and suggested that we will never have equity or parity in gender if we do not fight for racial justice and the rights of minorities.

Both Drs. Albright and Davis discussed the importance of context, locally, nationally, and globally. At a global health and healthcare level Dr. Davis recognized that given the many gaps in the US healthcare system (further highlighted by the COVID-19 pandemic), many parts of the US are having the same conversations we have historically had in LMICs. She went on to say that the ultimate solution is a comprehensive approach to care; although there may be different opportunities and challenges, fundamentally we are fighting for the same thing.

Dr. Albright shared her perspective in terms of global politics. ‘Disease knows no borders’ she said, ‘and therefore, there have to be some solutions to this crisis, and they have to happen internationally. Wherever we are, it affects what is happening to us at home. Therefore, we have become the perpetrators of our own victimization because we aren’t understanding our relationship to other countries.’ On a personal note, Dr. Albright also encouraged participants not to be so judgmental of each other, particularly in terms of the work-life decisions women make.

### Finding your passion and a worthy mission:

Many of our leaders emphasized the importance of a personal mission and core values for a successful career. Dr. Lola Adeyemi made a distinction between a social entrepreneur and an entrepreneur—it’s the mission. A social entrepreneur is a person who establishes an enterprise with the aim of solving social problems or affecting social change. While Dr. Adeyemi encouraged participants to ‘just go for [their ideas],’ she further noted the importance of learning from others, talking to people in the same space, and continuing to identify lessons learned. She said, ‘find people who do things you want to do…but do it better.’

When Dr. Mancuso spoke about her current role, she said, ‘I’m a nurse first, and a CEO and President second.’ Her goal is for all women to work together to learn about gender imbalance as well as gain the knowledge and skills to take on leadership assignments. She noted that her goal is ‘not to lose female talent, but to gain female talent…[and] to get [women’s] perspectives, get their knowledge, and bring it to the table.’ Connected to her point about life-long learning she urged participants to hold onto their core values and ‘learn, learn, learn.’

### Leadership skills

Two important but seemingly at-odds skills emerged as valuable for young women leaders. Speakers urged participants to ‘speak up,’ ‘take (or make) a seat at the table,’ and ‘be decisive.’ Many speakers shared their experiences in rooms full of men where they had to leverage their positions of authority and advanced degrees to be heard. Speakers also encouraged participants to listen more than they speak, building consensus, and admit when you don’t know something. This latter point is tightly connected to the value of life-long learning and building teams.

Most speakers lauded the value of so-called ‘soft’ or ‘essential skills’ (i.e., communication, self-awareness, team awareness), and saw these skills as great strengths among many women leaders. Dr. Albright also discussed the value of multi-tasking as an essential skill for leadership.

## Forming the Network

During the first session, participants called for the development of a dedicated networking space where they could engage with peers, learn about future events, and generally discuss women’s leadership issues. While this was not in the original scope of our work, the team felt this would provide substantial benefits to our participants, providing a space tailored to our participants’ needs. Within 1 day of the event, our team developed and invited all participants to Slack^©^, an online team workspace and communication tool designed specifically to facilitate such connections and conversations.

A set of 7 topical channels were created including general, mentorship, networking, seminar series, in search of job, in search of talent, and career resources. Responsive to new conversations and requests, this list evolved to include new channels for resource sharing (i.e., books and articles) and career transition (i.e., those coming back to global health after a hiatus).

Following each seminar, we developed an ‘afterparty’ or blocked time when a set of team members would be online, facilitating further conversations. To date we have hosted 3 afterparties, one of which was also facilitated by a speaker. Facilitated conversation channels are the most frequently used, followed by the general and networking channel, where participants are invited to share their LinkedIn profiles and information about their interests. At the time of writing this manuscript, the workspace had over 700 members.

**Figure 1:**
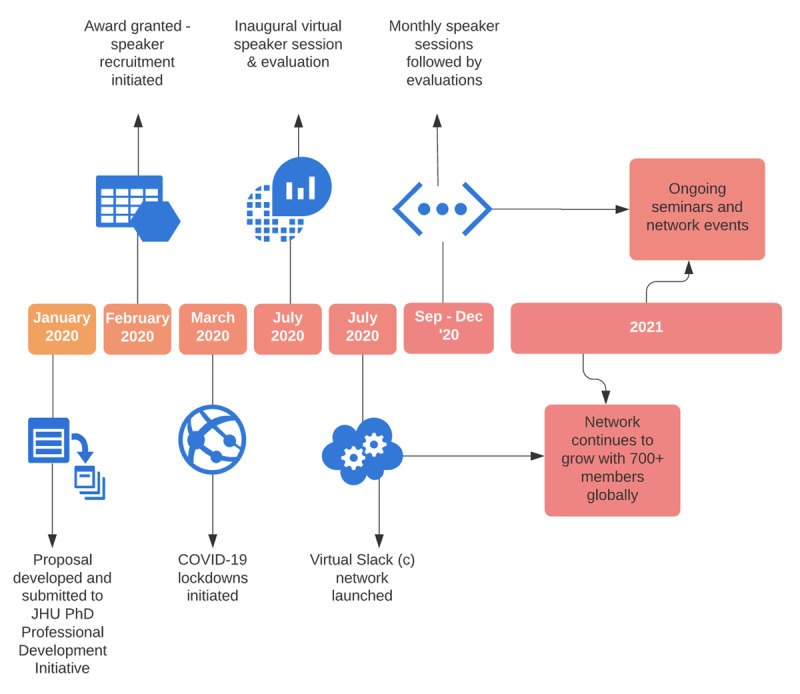
Timeline of Activities.

A timeline of activities is displayed in ***Figure 1***.

## Evaluation Results

Over the 5 seminars, we had 1,309 total seminar views, with an average of 268 attendees participating in each session. These sessions attracted 1,026 unique participants representing 44 countries. Of the participants, 605 (59%) were students at the time of attendance. Of those students, 266 (43.9%) were doctoral students, 296 (48.9%) were masters students, and 36 (6%) were undergraduate students. ***Table 1*** displays unique participant demographics.

**Table 1:** Participant Demographics.


REGIONS REPRESENTED	N = 1026	%

African Region AFRO	26	2.5

Eastern Mediterranean Region EMRO	5	0.4

European Region EURO	56	5.4

Region of the Americas PAHO	904	88.1

South-East Asia Region SEARO	25	2.4

Western Pacific Region WPRO	10	1.0

**PARTICIPANT STUDENT STATUS**	**N = 1026**	**%**

Student	605	59.0

Non-student	421	41.0

**STUDENT PARTICIPANT DEGREE PROGRAM**	**N = 605**	**%**

Doctoral student	266	43.9

Masters student	296	48.9

Undergraduate student	36	6.0

Other student	7	1.2


Eight hundred and seventy-six (85.3%) participants indicated that they were interested in pursuing or were currently working in a career in global health, while 116 (11.3%) were unsure. Seven hundred forty-nine (73%) participants were interested in pursuing or were currently working in a non-academic career.

Following each seminar, we conducted an evaluation using an online survey developed in Google Forms^©^. The tool included a mix of demographic, yes/no, 5-point Likert scale, and open-ended questions. Evaluations of the first two events were sent within 2 days of the event to the email that participants provided when they registered. A follow-up reminder was sent approximately one week after the event. Starting at the third event (and each subsequent event) we arranged for the evaluation to appear immediately after a participant left the event. Reminders were still sent after one week.

A total of 199 evaluations were completed during the series, representing a 15% response rate (ranging 10–22% after each seminar). On average, approximately 40 evaluations were completed per seminar (range: 27–75). ***Table 2*** displays the evaluation results.

**Table 2:** Participant Evaluation Results.


LIKERT SCALE ITEM (N = 199)	STRONGLY AGREE	AGREE	NEITHER	DISAGREE	STRONGLY DISAGREE

The content of the seminar was relevant to my interests as a participant	98 (49.5)	71 (35.9)	8 (4.0)	2 (1.0)	20 (10.1)

I have a greater understanding of solutions to combat challenges that women face in the field of global health leadership	43 (21.7)	70 (35.4)	55 (27.8)	16 (8.1)	15 (7.6)

I have a better understanding of different career paths in global health	79 (39.9)	78 (39.4)	16 (8.1)	7 (3.5)	19 (9.6)

Based on this event, I would attend the next event in the series	118 (59.6)	55 (27.8)	6 (3.0)	0 (0)	20 (10.1)

**EVALUATION QUESTIONS**	**YES (%)**	**NO (%)**	**UNSURE (%)**		

This event strengthened my network opportunities	107 (53.7)	26 (13.0)	66 (33.2)		

I would recommend the series to others	197 (99.0)	0 (0)	2 (1.0)		


Among the respondents, 49.5% (n = 98) strongly agreed and 35.9% (n = 71) agreed that the content of the seminar was relevant to their interests as a participant; 39.9% (n = 79) strongly agreed and 39.4% (n = 78) agreed that they have a greater understanding of solutions to combat challenges that women face in the field of global health leadership. Twenty-one point seven percent (n = 43) strongly agreed and 35.4% (n = 70) agreed that they have a better understanding of different career paths available to them in global health after the seminar.

Fifty-three point seven percent (n = 107) of respondents reported that the event strengthened their network; 59.6% (n = 118) strongly agreed and 27.8% (n = 55) agreed that based on the event they attended they would attend the next event in the series. Ninety nine percent (n = 197) of respondents indicated they would recommend the series to others.

Open-ended questions were used to elicit additional feedback about each session and suggestions for future directions. Some participants found the online chat distracting and suggested we allot dedicated time at the beginning of each session for people to introduce themselves, share contact information, and network; one participant suggested we move all networking activities to our Slack workspace. A few participants requested more information about the workspace before joining and indicated they were less familiar with the platform and its functions. Some participants also requested further interaction opportunities with the speakers in addition to the moderated Q&A period. Finally, some respondents called for greater diversity in speakers and requested we highlight leaders with varying levels of experience (i.e., recent graduates) and credentials (i.e., Master’s or Bachelor’s degrees).

## Informing Leadership Development Programming

Many events, conferences, and programs designed to support women leaders in global health are developed by and feature senior leaders. WomenLift Health convenes the annual Women Leaders in Global Health (WLGH) conference, featuring more than 2,000 attendees from approximately 50 countries [16]. Women in Dev is a platform which connects women working in international development and advocates for feminist leadership models, transformed funding practices, and an increase in women’s leadership [17]. Academic institutions are increasingly offering women’s leadership programs that include fellowships, skills-based training, coaching, and mentorship.

While many leaders featured in our series were also senior, we explored the additional value of peer and near-peer components through the working groups and additional online networking spaces. Senior leaders may be likely to draw larger audiences (as we saw with Dr. Madeleine Albright) but there is distinct value in focusing on the development of more proximal relationships. In fact, many of our speakers described the value of expanding networks and finding near-peer mentors. Based on the active participation in the sessions and networking space we see this as an area of need and growth in the women’s leadership space.

We believe the pandemic provided an opportunity to recruit high-level speakers whose schedules may have otherwise posed a significant challenge. Moving the events online freed up substantial portions of the budget originally intended to support speaker travel and event catering. These resources allowed us to place greater emphasis on communications and outreach to doctoral students and young professionals beyond the immediate JHU network; this yielded a much broader audience that to date has represented 44 countries. Given the high levels of attendance and consistent participation in the network, there is clearly an appetite for women’s leadership programming.

Academic institutions have an opportunity to capitalize on this interest to bolster leadership skills among their students. While many institutions offer an array of services and resources already (including career services, mentorship programs, and a built-in alumni network), participants from our working group noted that it is time consuming to keep track of the many opportunities while balancing competing priorities. Leadership programs should collate and curate such resources for interested students which could bolster networking opportunities both within and outside the institution. Mentors may be expected to direct students to resources but it has been established that mentors are often overextended and under supported [18]. Our working group similarly noted that mentors need more institutional support including protected time to mentor and training resources on how to effectively mentor.

Doctoral curriculums are increasingly called upon to foster professional development and leaderships skills in addition to the substantive and methodological training that historically would have constituted the sole content. This program’s combination of high-level speakers with opportunity for reflection, coupled with focused sustained online discussion forums among students, lends itself readily to inclusion within doctoral training curricula and offers the additional advantage of building strong global professional networks spanning peers, near-peers, and more advanced stages of the career trajectory. This type of programming can also support a diverse cadre of women leaders, including those with intersecting identities that are often marginalized or historically invisible in leadership ranks, e.g., on the basis of race/ethnicity or socio-economic status—so-called academic ‘first gens.’

While this program launched with nominal resources, it requires more financial and personnel support as it grows. Institutions should consider and plan for the costs of events (i.e., communications, honoraria for speakers often asked to donate their time, and dedicated support for personnel) and network maintenance (again, time and dedication). We also believe it is important for such programs to have a mission driving it forward, to amplify diverse voices, and to take a solutions-oriented approach that develops materials to be used for advocacy and institutional and individual growth.
